# A dose-escalation study of indisulam in combination with capecitabine (Xeloda) in patients with solid tumours

**DOI:** 10.1038/sj.bjc.6604300

**Published:** 2008-04-15

**Authors:** W S Siegel-Lakhai, A S Zandvliet, A D R Huitema, M M Tibben, G Milano, V Girre, V Diéras, A King, E Richmond, J Wanders, J H Beijnen, J H M Schellens

**Affiliations:** 1 Department of Pharmacy & Pharmacology, The Netherlands Cancer Institute/Slotervaart Hospital, Amsterdam, The Netherlands; 2 Oncopharmacology Unit, Centre Antoine Lacassagne, Nice, France; 3 Department of Medical Oncology, Institut Curie, Paris, France; 4 Eisai Ltd., London, UK; 5 Department of Biomedical Analysis, Section of Drug Toxicology, Utrecht University, Utrecht, The Netherlands; 6 Department of Medical Oncology, The Netherlands Cancer Institute/Antoni van Leeuwenhoek Hospital, Amsterdam, The Netherlands

**Keywords:** indisulam, capecitabine, phase I, pharmacokinetics, drug–drug interaction

## Abstract

This dose escalation study was designed to determine the recommended dose of the multi-targeted cell cycle inhibitor indisulam in combination with capecitabine in patients with solid tumours and to evaluate the pharmacokinetics of the combination. Thirty-five patients were treated with indisulam on day 1 of each 21-day cycle. Capecitabine was administered two times daily (BID) on days 1–14. Plasma concentrations of indisulam, capecitabine and its three metabolites were determined for pharmacokinetic analysis. The main dose-limiting toxicity was myelosuppression. Hand/foot syndrome and stomatitis were the major non-haematological toxicities. The recommended dose was initially established at indisulam 700 mg m^−2^ and capecitabine 1250 mg m^−2^ BID. However, during cycle 2 the recommended dose was poorly tolerated in three patients. A dose of indisulam 500 mg m^−2^ and capecitabine 1250 mg m^−2^ BID proved to be safe at cycle 1 and 2 in nine additional patients. Indisulam pharmacokinetics during cycle 1 were consistent with pharmacokinetic data from phase I mono-therapy studies. However, exposure to indisulam was remarkably increased at cycle 2 due to a drug–drug interaction between capecitabine and indisulam. Partial response was confirmed in two patients, one with colon carcinoma and the other with pancreatic carcinoma. Seventeen patients had stable disease. Indisulam (700 mg m^−2^) in combination with capecitabine (1250 mg m^−2^ BID) was well tolerated during the first cycle. A dose of indisulam 500 mg m^−2^ and capecitabine 1250 mg m^−2^ BID was considered safe in multiple treatment cycles. The higher incidence of toxicities observed during cycle 2 can be explained by a time-dependent pharmacokinetic drug–drug interaction.

Deregulation of the cell cycle commonly occurs during tumour development, resulting in unrestricted cell proliferation (Sherr, 1996; Lundberg and Weinberg, 1999; Owa *et al*, 2001). Cyclin-dependent kinases (CDKs) in concert with cyclin proteins control the progression of the cell cycle. CDK inhibitors have the potential to induce cell cycle arrest and apoptosis in tumour cells. Indisulam is a chloro-indoyl sulphonamide anticancer agent that inhibits the phosphorylation of CDK2 and decreases the expression of cyclin E (Ozawa *et al*, 2001). This is accompanied by hypophosphorylation of the retinoblastoma protein. Indisulam also reduces the expression of cyclins A, B1 and H. This ultimately results in cell cycle arrest at multiple checkpoints. At higher concentrations, treatment with indisulam was associated with upregulation of p53 and p21 resulting in apoptotic cell death (Fukuoka *et al*, 2001; Ozawa *et al*, 2001).

Potent tumour growth inhibition has been demonstrated in both *in vitro* and *in vivo* models (Ozawa *et al*, 2001).

Indisulam was clinically evaluated in five phase I studies in patients with refractory solid tumours and in several phase II studies including patients with colorectal cancer, breast cancer, head and neck cancer, NSCLC, renal cell cancer and metastatic melanoma (Raymond *et al*, 2000, 2002; Punt *et al*, 2001; Mainwaring *et al*, 2002; Dittrich *et al*, 2003; Fumoleau *et al*, 2003; Terret *et al*, 2003; Haddad *et al*, 2004; Raftopoulos *et al*, 2004; Smyth *et al*, 2005; Yamada *et al*, 2005; Talbot *et al*, 2007). Reversible neutropenia and thrombocytopenia were the dose-limiting toxicities in all schedules. The maximum-tolerated dose (MTD) of indisulam was established at 800 mg m^−2^ when given as a one-hour infusion once every 3 weeks (Raymond *et al*, 2002). A dose of 700 mg m^−2^ was considered safe for further studies and was evaluated in the phase II programme (Mainwaring *et al*, 2002; Fumoleau *et al*, 2003; Haddad *et al*, 2004; Raftopoulos *et al*, 2004; Smyth *et al*, 2005; Talbot *et al*, 2007). Although indisulam was well tolerated, it had only minor to moderate single agent activity in several phase II studies (Mainwaring *et al*, 2002; Fumoleau *et al*, 2003; Haddad *et al*, 2004; Raftopoulos *et al*, 2004; Smyth *et al*, 2005; Talbot *et al*, 2007).

Currently, the combination of indisulam with several classes of antineoplastic drugs is being investigated. The combination of indisulam with capecitabine showed additive effects in preclinical studies when both agents were given simultaneously or when indisulam was given prior to capecitabine, which might be explained by downregulation of thymidylate synthetase by indisulam (Ozawa *et al*, 2004). On the basis of the preclinical data, the different mechanisms of antitumor activity of indisulam and capecitabine and the largely non-overlapping toxicity profiles, the current phase I trial was performed with this combination. The primary objectives were: (i) to determine the recommended dose and (ii) to evaluate the pharmacokinetic profiles of indisulam and capecitabine. An additional objective was to explore preliminary activity of this combination.

## PATIENTS AND METHODS

### Eligibility

Patients were eligible if they had a histologically or cytologically confirmed solid tumour refractory to standard therapy or for whom no established therapy existed. Previous anticancer radiotherapy or chemotherapy had to be discontinued for at least 4 weeks before entry into the study. Prior capecitabine therapy was allowed, but a severe or unexpected reaction to fluoropyrimidine therapy was an exclusion criterion. Maximal two prior lines of myelosuppressive chemotherapy were allowed. Patients had to have acceptable bone marrow, renal and hepatic functions. The study protocol was approved by the medical ethics committees of the participating hospitals and all patients gave written informed consent (World Medical Association Declaration of Helsinki, 2002).

### Treatment plan and study design

The recommended dose of indisulam in combination with capecitabine was determined by dose escalation. Patients were initially treated in cohorts of three per dose level. Each cycle consisted of 3 weeks of therapy. The starting doses were 350 mg m^−2^ of indisulam given as a 1-h infusion on day 1 and 1000 mg m^−2^ BID of capecitabine given on days 1–14, repeated 3 weekly. On day 1, the start of the infusion of indisulam and the oral administration of capecitabine were simultaneous. In the next cohort, the dose of capecitabine was escalated to 1250 mg m^−2^ BID, which is the recommended dose for treatment of colon, colorectal and breast cancer. In subsequent cohorts, the dose of indisulam was escalated to 500, 600, 700 and 800 mg m^−2^ when permitted by the toxicity profile of the combination. If one of the first three patients experienced dose-limiting toxicity (DLT) during cycle 1, the cohort was expanded to six patients. Provided that none of the additional three patients experienced a DLT during cycle 1, dose escalation was continued. Patients who did not experience significant toxicities at their initial dose were permitted to receive a dose escalation. The MTD was defined as the dose level at which two or more patients in the expanded cohort of six patients experienced a DLT. The dose level below the MTD was recommended for phase II evaluation.

### Patient evaluation and follow-up

Complete patient history, physical examination, performance status, haematological analysis, blood chemistry and urinalysis were performed at baseline. Electrocardiogram (ECG) recordings were taken pre-dose and within 1 h after the end of the indisulam infusion in cycle 1. Computed tomography, magnetic resonance imaging scans or photography for skin lesions were performed to clearly document the location, size and extent of the disease. Laboratory tests were performed on day 1, 8 and 15 of each treatment cycle. Tumour assessments were performed every other cycle and were evaluated according to the RECIST criteria (Therasse *et al*, 2000). Adverse events were evaluated throughout the study and were graded according to the National Cancer Institute Common Toxicity Criteria version 2.0 (Cancer Therapy Evaluation Program, 1998).

DLT was defined as: grade 3 or 4 non-haematological toxicity (excluding alopecia and untreated nausea and vomiting), grade 4 thrombocytopenia, grade 4 neutropenia lasting 7 days or more and grade 3 or 4 febrile neutropenia. These events were indicated as DLT only if observed during the first cycle of treatment or during cycle 2 for patients who underwent intra-patient dose escalation.

### Pharmacokinetic study

Full pharmacokinetic sampling of indisulam and capecitabine were performed during the first cycle of treatment. For patients who underwent a dose escalation at cycle 2, full pharmacokinetic sampling was repeated in the first cycle at the increased dose. Blood samples for indisulam analysis were obtained at 16 time points for up to 8 days after the first administration: pre-infusion, 30 min after the start of infusion, at the end of the infusion, at 10 and 30 min and at 1, 2, 4, 6, 8, 24, 48, 72, 96, 120 and 168 h after the end of the infusion. The concentrations of indisulam in plasma were measured using high-performance liquid chromatography coupled to an electrospray ionisation tandem mass spectrometer (LC/ESI-MS/MS) as described previously (Beumer *et al*, 2004). Blood samples for capecitabine analysis were obtained at 10 time points on day 1: before administration, at 15 and 30 min and at 1, 2, 3, 4, 5, 7 and 9 h after administration. Plasma concentrations of capecitabine and its metabolites 5′-deoxy-5-fluorocytidine (5′-DFCR), 5′-deoxy-5-fluorouridine (5′-DFUR) and 5-fluorouracil (5-FU) were determined by high-performance liquid chromatography with UV detection (Reigner *et al*, 1998).

### Population pharmacokinetic analysis

The population pharmacokinetic analyses were performed using NONMEM software (version V, level 1.1) (GloboMax LLC, Hannover, USA) (Beal *et al*, 1988). A population pharmacokinetic model was developed previously to describe the pharmacokinetic profile of indisulam mono-therapy (Zandvliet *et al*, 2006). The pharmacokinetic parameters describing the model for indisulam mono-therapy were applied to calculate model predicted indisulam concentrations, which were compared to the observed plasma concentration of indisulam. If the model adequately predicted the observed concentrations, it could be concluded that the pharmacokinetic profile of indisulam was not highly influenced by combination therapy with capecitabine.

The pharmacokinetic results of capecitabine and its metabolites were also evaluated by compartmental analysis using NONMEM software to adequately describe the absorption phase. A time delay was observed between drug intake and the appearance of capecitabine in plasma. Therefore, a first-order absorption model with lag-time was applied to all data. Individual areas under the concentration *vs* time curve (AUCs) and terminal half-lives were assessed by fitting a one- or two-compartment model to the concentration time profiles in individual patients. The current data were compared to previously published results to evaluate any potential pharmacokinetic interaction with indisulam.

### Pharmacokinetic–pharmacodynamic analysis

The correlation between drug exposure and CTC grade 3 and 4 adverse events was explored to find potential relationships between pharmacokinetics and safety parameters. The AUC was used as a measure of exposure to indisulam in this analysis. For capecitabine, the AUC of 5′-DFUR was previously found to be predictive of toxicities and was therefore used in the current analysis to assess relationships between exposure to capecitabine and adverse events (Gieschke *et al*, 1998).

## RESULTS

### Dose escalation and safety assessment

In total, 35 patients were included in the study. Patient characteristics are shown in Table 1. All patients were evaluable for toxicity. Patients were initially treated at six different dose-levels (level 1–6) (Table 2a). The number of treatment cycles per patient (median=3, range 1–15) is summarised in Table 2b. In total, patients received 159 cycles.

Dose levels 1, 2 and 3 were well tolerated. A patient who had an intra-patient dose escalation from level 3 to level 4 experienced dose limiting toxicity at the escalated dose: febrile neutropenia grade 3 and thrombocytopenia grade 3. Another patient with thymoma died due to cardiac arrhythmia during indisulam infusion at cycle 1. It could not be precluded that this serious adverse event was related to study medication. For safety reasons, patients at level 4 were further treated at level 3. Two additional patients were treated at dose level 3. Normal dose escalation was resumed and continued up to dose level 6 (800 mg m^−2^ indisulam).

The first two patients treated at dose level 6 both experienced serious haematological toxicity. One patient had febrile neutropenia grade 3, neutropenia grade 4, leukocytopenia grade 3 and thrombocytopenia grade 3 and the other patient had febrile neutropenia grade 4, leukocytopenia grade 4, thrombocytopenia grade 3 and neutropenia grade 4. Because of safety reasons, it was decided not to further expand dose level 6 and this dose level was established as the MTD.

At the immediate lower dose level of 700 mg m^−2^ indisulam in combination with 1250 mg m^−2^ BID capecitabine, four patients were additionally treated. This level was well tolerated at cycle 1. Conversely, all four patients experienced severe toxicities during cycle 2. Three patients had severe haematological and non-haematological toxicities at cycle 2 and consequently dose reduction of both drugs; all three patients had neutropenia grade 4, thrombocytopenia grade 3 or 4, leukocytopenia grade 3 or 4 and hand/foot reaction grade 3 or 4, and one of these patients also had febrile neutropenia grade 3, anaemia grade 3 and stomatitis grade 3. The fourth patient had stomatitis grade 3 and treatment with capecitabine was interrupted.

The significant toxicities observed after multiple administrations of dose level 5 indicated that this recommended dose level was safe during cycle 1, but was too toxic for repeated cycles. Therefore, the dose of indisulam was further reduced to 500 mg m^−2^ (dose level 7, Table 2a) and an amended study was initiated to investigate safety at repeated cycles. To investigate a potential time-dependent pharmacokinetic interaction between indisulam and capecitabine, the additional patients had full pharmacokinetic sampling of indisulam and capecitabine during cycle 1 and 2 and were assessed for DLT at both cycles. In this amended study, nine eligible patients were treated with 500 mg m^−2^ indisulam in combination with 1250 mg m^−2^ BID capecitabine. One patient had dose-limiting stomatitis grade 3 at cycle 1 and another patient had dose-limiting hand/foot reaction grade 3 during treatment cycle 2. Generally, this dose level was adequately tolerated and was considered safe for multiple treatment cycles.

**Figure 1:**
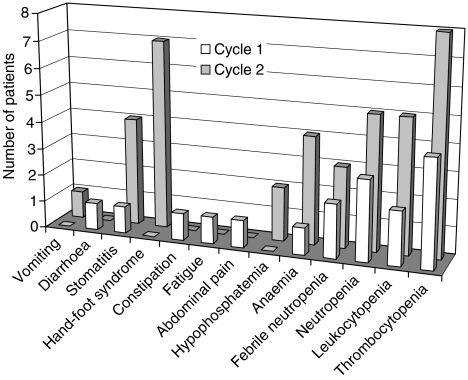
Graphical representation of severe (grade 3 and 4) adverse events in cycle 1 and in cycle 2.

Tables 3a and 3b show a summary of treatment-related adverse events. Haematological and non-haematological toxicities were observed more frequently at higher dose levels. The most frequent grade 3–4 haematological toxicities related to study treatment during cycle 1 were thrombocytopenia (four patients) and neutropenia (three patients) (Table 3a). Two patients had febrile neutropenia during cycle 1. The most frequent non-haematological toxicities related to study treatment were stomatitis (12 patients), diarrhoea (10 patients), hand-foot syndrome (nine patients) and nausea (nine patients) (Table 3b). The occurrence of severe adverse events (CTC grade 3 and 4) in cycle 1 and in cycle 2 is depicted in Figure 1. This bar chart demonstrates that severe adverse events were less frequently observed in cycle 1 than in cycle 2.

The main grade 1–2 adverse events were anaemia, diarrhoea, nausea and vomiting (Table 3b). ECG analysis showed that indisulam did not have any cardiac effect.

A total of 16 patients (46%) went off-study due to progressive disease according to RECIST criteria, seven (20%) due to clinical progression, seven (20%) due to adverse events, three (8.6%) because of no further clinical benefit (physicians decision), one patient because the delay between treatment cycles was greater than 2 weeks (protocol deviation), and one because of an abnormal bilirubin level grade 3. Seven patients (20%) died on study, defined as within 30 days of the last intake of study treatment.

### Response

Thirty patients were evaluable for tumour response. Five patients (14%) could not be evaluated because they did not receive at least two cycles of treatment. Two patients had confirmed partial response (6%), 17 patients (49%) had stable disease and 11 patients (31%) showed disease progression.

### Population pharmacokinetics

Plasma samples for pharmacokinetic studies were obtained from 33 patients at cycle 1 for both indisulam and capecitabine. Two patients were not evaluable for pharmacokinetic assessment. At cycle 2, additional pharmacokinetic sampling was performed in eight patients. For indisulam, in total 573 pharmacokinetic samples were available from 41 treatment cycles (14 per cycle). During cycle 1, the previously developed population pharmacokinetic model adequately described the non-linear pharmacokinetic profile of indisulam. Model-based predictions corresponded well to the observed indisulam concentrations at cycle 1 (data not shown). No difference was observed between pharmacokinetic data from a phase I mono-therapy study and cycle 1 of the current combination study. This indicates that capecitabine does not interact with indisulam pharmacokinetics at treatment cycle 1.

**Figure 2:**
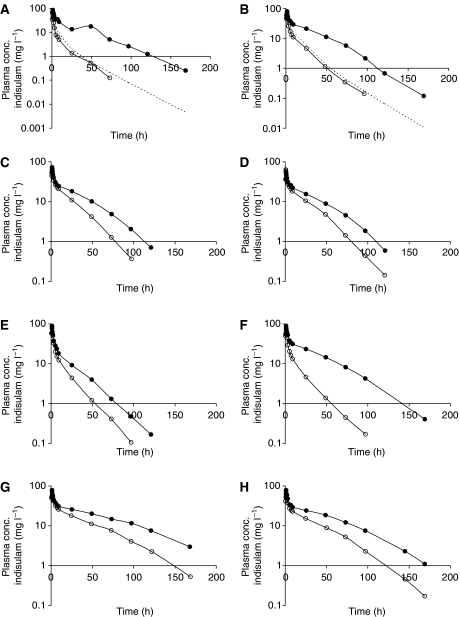
Observed indisulam plasma concentrations at cycle 1 (○) and cycle 2 (•) for eight individual patients. Patients **A** and **B** received an escalated indisulam dose at cycle 2. The broken line (- - - -) represents the expected concentration time profile if capecitabine did not interact with indisulam pharmacokinetics. Patients **C**, **D**, **E**, **F**, **G** and **H** were included in the amended study and received both in cycle 1 and in cycle 2 500 mg m^−2^ indisulam.

However, plasma concentrations of indisulam at cycle 2 were much higher than expected. This is illustrated in Figure 2. Patient A and B were treated during the initial part of the study. Patient A was treated with 350 mg m^−2^ of indisulam (level 2) at cycle 1 and the corresponding AUC was 0.21 g l^−1^ h^−1^. Assuming that the individual pharmacokinetic parameters would not be significantly different at cycle 2, the AUC after administration of the second indisulam infusion (level 3) was expected to be 0.32 g l^−1^ h^−1^ for this patient. However, the observed AUC was 1.51 g l^−1^ h^−1^. For patient B, the AUC after initial treatment with 500 mg m^−2^ indisulam (level 3) was 0.42 g l^−1^ h^−1^ and the expected AUC value for cycle 2 (level 4) was 0.53 g l^−1^ h^−1^. However, the observed AUC of 1.51 g l^−1^ h^−1^ was again much higher than expected, suggesting a time-dependent pharmacokinetic interaction between indisulam and capecitabine. This was confirmed in the amended study. Without exception, six additional patients (level 7) were much higher exposed to indisulam during cycle 2 than during cycle 1.

For capecitabine and its metabolites, 883 samples were available from 33 patients. Pharmacokinetic results are summarised in Table 4. Data from the current study were compared to literature data (Mackean *et al*, 1998; Reigner *et al*, 1998, 1999; Cassidy *et al*, 1999; Twelves *et al*, 1999). The exposure to capecitabine and its metabolites in the current study was not statistically significantly different from published data.

### Pharmacokinetic–pharmacodynamic analysis

Severe adverse events (CTC grade 3 and 4) occurred in 11 out of 41 treatment cycles, which were assessed for pharmacokinetics. The AUC of indisulam was significantly higher in these 11 cycles (median 2.2 g l^−1^ h^−1^, range 0.7–5.7 g l^−1^ h^−1^) than in the remaining 30 cycles (median 1.1 g l^−1^ h^−1^, range 0.2–4.3 g l^−1^ h^−1^) (Mann–Whitney *U* test, *P*=0.008). This indicates that high exposure to indisulam was associated with higher risk of severe adverse events. The highest exposure to indisulam (5.7 g l^−1^ h^−1^) was observed in a patient who had dose-limiting neutropenia with sepsis, leukocytopenia grade 4 and thrombocytopenia grade 3. Exposure to the capecitabine metabolite 5′-DFUR was not higher in treatment cycles with grade 3 and 4 toxicities (Mann–Whitney *U* test, *P*=0.48).

## DISCUSSION

In this study, treatment with indisulam in combination with capecitabine was investigated in patients with solid tumours. We demonstrated that 500 mg m^−2^ indisulam and 1250 mg m^−2^ BID capecitabine is safe and well tolerated during multiple cycles.

The recommended dose for indisulam single agent therapy was 700 mg m^−2^ (Raymond *et al*, 2002). In this study, the combined dose of 700 mg m^−2^ indisulam and 1250 mg m^−2^ BID capecitabine was well tolerated during cycle 1. However, at cycle 2 and subsequent cycles significant toxicities were observed, which indicated that this dose level was too toxic for repeated cycles. Therefore, the dose of indisulam was further reduced to 500 mg m^−2^ to investigate safety at multiple cycles. Of nine eligible patients, only one patient experienced a dose-limiting toxicity, which confirmed that 500 mg m^−2^ indisulam and 1250 mg m^−2^ BID capecitabine is safe for multiple treatment cycles.

Two patients had partial response and 17 patients had stable disease. Whether the combination of indisulam and capecitabine has a clinical additive effect remains to be investigated in phase II and III studies.

One subject died due to cardiac arrhythmia. However, ECG recordings in all other subjects did not show cardiac effects. Furthermore, in the extensive clinical development of indisulam, no signs of increased risk on cardiac toxicity was noticed. Continuous cardiac monitoring is therefore not deemed necessary in future clinical trials.

The main dose-limiting toxicity was myelosuppression. Grade 3–4 non-haematological toxicities most frequently observed were hand/foot reaction and stomatitis. Hand/foot reaction is dependent on cumulative exposure to capecitabine (Hénin *et al*, 2006). The incidence of this adverse event was therefore expected to be higher at cycle 2 than at cycle 1. However, the increased incidence of other grade 3–4 toxicities was suggestive for a time-dependent drug–drug interaction between indisulam and capecitabine.

The exposure to indisulam was highly increased in cycle 2, which is likely due to a pharmacokinetic interaction with capecitabine. It has been postulated that 5-FU, the active metabolite of capecitabine, may interfere with the synthesis of the cytochrome P450 isozyme CYP2C9 (Brown, 1999). This isozyme is mainly responsible for the metabolism of indisulam (Eisai Ltd., data on file). Thus, through inhibition of CYP2C9 synthesis, indisulam metabolism may be impaired. This same type of interaction has been described for phenytoin and warfarin in combination with 5-FU (Copur *et al*, 2001; Gilbar and Brodribb, 2001). Phenytoin and warfarin are also metabolised by CYP2C9. In combination with 5-FU the clearances of these agents were decreased and plasma concentrations were increased. As 5-FU downregulates the synthesis of CYP2C9 (Brown, 1999; Copur *et al*, 2001; Gilbar and Brodribb, 2001), it is not surprising that this interaction becomes relevant only during the second or subsequent treatment cycles of the combination capecitabine and indisulam. The results suggest that CYP2C9 activity is decreased even after a 7-day dosing interval of capecitabine. This finding is in agreement with the results of Konishi *et al* (2003), who demonstrated that the decrease in phenytoin p-hydroxylation after single doses of 5-FU or 5′-DFUR was not fully resolved after 7 days.

Owing to a time-dependent pharmacokinetic interaction between the drugs, patients were exposed to higher concentrations of indisulam during cycle 2 than during cycle 1 (Zandvliet *et al*, 2007). Accordingly, the incidence of severe toxicities was higher in repeated treatment cycles. A dose of indisulam of 500 mg m^−2^ and capecitabine 1250 mg m^−2^ BID is considered safe in multiple treatment cycles. Future *in vitro* and animal studies may further elucidate the mechanisms of the time-dependent interaction between indisulam and capecitabine.

## Figures and Tables

**Table 1:** Patient characteristics at screening

	***N*=35**
*Gender*	
Male	22 (63%)
Female	13 (37%)
	
*Age*	
Median	56
Range	20–70
	
*Race*	
Caucasian	35 (100%)
	
*Tumour type*	
Pancreas	10 (29%)
ACUP	5 (14%)
Colon	5 (14%)
Stomach	3 (9%)
Rectum	2 (6%)
NSCLC	2 (6%)
Oesophagus	1 (3%)
Ewing bone sarcoma	1 (3%)
Sclerosing osteosarcoma	1 (3%)
Melanoma	1 (3%)
Cholangiocarcinoma	1 (3%)
Squamous cell carcinoma	1 (3%)
Thymoma	1 (3%)
Endometrium	1 (3%)
	
*KPS performance status*	
70	14 (40%)
80	10 (29%)
90	8 (23%)
100	3 (9%)
	
*Time since diagnosis (months)*	
Median	11
Range	0–108
	
*Metastatic/locally advanced disease*	
Metastatic	30 (86%)
Locally advanced	5 (14%)
	
*Previous therapy*	
Chemotherapy	22 (63%)
Radiotherapy	12 (34%)
Surgery	23 (66%)
Other	4 (11%)

**Table 2a:** Doses of indisulam and capecitabine and number of patients included per dose level

**Dose level**	**Indisulam (mg m^−2^) day 1**	**capecitabine (mg m^−2^) BID days 1–14**	***N***
1	350	1000	3
2	350	1250	4
3	500	1250	6
4	600	1250	4
5	700	1250	7
6	800	1250	2
7	500	1250	9

**Table 2b:** Number of cycles per patient

**Number of cycles**	***N***
1–3	20 (57%)
4–6	7 (20%)
>6	8 (23%)

**Table 3a:** Treatment-related NCI-CTC grade 3–4 hematological lab values during cycle 1

**Dose level**	**1**	**2**	**3 and 7**	**4**	**5**	**6**	**Total**
**number of patients treated**	***n*=3**	***n*=4**	***n*=15**	***n*=4**	***n*=7**	***n*=2**	***n*=35**
Thrombocytopenia	0	0	1	0	1	2	4 (11%)
Neutropenia	0	0	1	0	0	2	3 (9%)
Leukocytopenia	0	0	0	0	0	2	2 (6%)
Anaemia	0	0	0	0	1	0	1 (3%)

**Table 3b:** Treatment-related non-haematological adverse events (observed in at least two patients) during cycle 1

**Dose level**	**1**		**2**		**3 and 7**		**4**		**5**		**6**		**Total**	
**number of patients treated**	***n*=3**		***n*=4**		***n*=15**		***n*=4**		***n*=7**		***n*=2**		***n*=35**	
**NCI-CTC grade**	**1–2**	**3–4**	**1–2**	**3–4**	**1–2**	**3–4**	**1–2**	**3–4**	**1–2**	**3–4**	**1–2**	**3–4**	**1–2**	**3–4**
Stomatitis	—	—	—	—	5	1	1	—	3	—	2	—	11 (31%)	1 (3%)
Diarrhoea	—	—	2	—	3	1	1	—	3	—	—	—	9 (26%)	1 (3%)
Hand-foot syndrome	—	—	—	—	4	—	2	—	2	—	1	—	9 (26%)	0 (0%)
Nausea	—	—	1	—	3	—	—	—	4	—	1	—	9 (26%)	0 (0%)
Vomiting	—	—	1	—	2	—	1	—	2	—	1	—	7 (20%)	0 (0%)
Fatigue	—	—	—	—	3	—	—	—	—	1	—	—	3 (9%)	1 (3%)
Abdominal pain	—	—	—	—	1	—	—	—	1	1	1	—	3 (9%)	1 (3%)
Oedema	—	—	1	—	—	—	—	—	2	—	—	—	3 (9%)	0 (0%)
Alopecia	—	—	—	—	—	—	—	—	1	—	2	—	3 (9%)	0 (0%)
Anorexia	—	—	—	—	1	—	—	—	2	—	—	—	3 (9%)	0 (0%)
Constipation	—	—	—	—	—	1	1	—	—	—	1	—	2 (6%)	1 (3%)
Epistaxis	—	—	—	—	2	—	—	—	—	—	—	—	2 (6%)	0 (0%)
Dyspepsia/pyrosis	—	—	—	—	2	—	—	—	—	—	—	—	2 (6%)	0 (0%)
Flatulence/meteorism	—	—	—	—	—	—	—	—	2	—	—	—	2 (6%)	0 (0%)
Hiccups	—	—	—	—	2	—	—	—	—	—	—	—	2 (6%)	0 (0%)

For events not included in NCI-CTC: mild=grade 1; moderate=grade 2; severe=grade 3.

**Table 4:** Summary of pharmacokinetic analysis of capecitabine and its metabolites 5′-DFCR, 5′-DFUR and 5-FU

		**Capecitabine**	**5′-DFCR**	**5′-DFUR**	**5-FU**
Number of patients evaluable for PK assessment		32	32	32	33
Number of curves evaluable for PK assessment^a^		40	40	40	41
Number of curves fitted to a 1-compartment model		29	26	35	35
Number of curves fitted to a 2-compartment model		11	14	5	6
					
Absorption rate constant of capecitabine (h^−1^)	mean^b^	5.27			
	CV^b^ (%)	248%			
Formation rate constant of metabolite (h^−1^)	mean^b^		2.60	3.71	3.96
	CV^b^ (%)		139%	170%	154%
Absorption lag time of capecitabine (h)	mean^b^	0.57			
	CV^b^ (%)	109%			
Formation lag time of metabolite (h)	mean^b^		0.53	0.74	0.73
	CV^b^ (%)		101%	90%	111%
Terminal half-life (h)	mean^b^	0.73	0.30	1.03	0.83
	CV^b^ (%)	646%	852%	47%	320%
AUC (mg l^−1^ h^−1^)	mean^b^	7.71	12.5	9.36	1.29
Normalized AUC^c^ (mg l^−1^ h^−1^)	mean^b^	6.67	10.8	8.09	0.98
	CV^b^ (%)	91%	99%	40%	65%

a Eight patients were assessed for capecitabine pharmacokinetics during two cycles.

b Geometric means and geometric coefficients of variance (interindividual variability) are reported.

c AUC values were normalised to 2000 mg doses of capecitabine.
